# Therapeutic response monitoring after targeted therapy in an orthotopic rat model of hepatocellular carcinoma using contrast-enhanced ultrasound: Focusing on inter-scanner, and inter-operator reproducibility

**DOI:** 10.1371/journal.pone.0244304

**Published:** 2020-12-23

**Authors:** Hwaseong Ryu, Jung Hoon Kim, Seunghyun Lee, Joon Koo Han

**Affiliations:** 1 Department of Radiology, Pusan National University Yangsan Hospital, Yangsan-si, Gyeongsangnam-do, Korea; 2 Department of Radiology, Seoul National University Hospital, Jongno-gu, Seoul, Korea; 3 Department of Radiology, Seoul National University College of Medicine, Jongno-gu, Seoul, Korea; 4 Institute of Radiation Medicine, Seoul National University Medical Research Center, Jongno-gu, Seoul, Korea; Medical University of Vienna, AUSTRIA

## Abstract

**Purpose:**

To assess therapeutic response monitoring after targeted therapy in an orthotopic rat model of hepatocellular carcinoma (HCC) using CEUS with focusing on inter-scanner and inter-operator reproducibility.

**Materials and methods:**

For reproducibility, CEUS was performed using two different US scanners by two operators in sixteen rat models of HCC. Using perfusion analysis software (VueBox ®), eleven parameters were collected, and intra-class correlation coefficient (ICC) was used to analyze reproducibility. Then seventeen rat models of HCC were divided into treatment group (n = 8, 30 mg/kg/day sorafenib for five days) and control group (n = 9). CEUS was performed at baseline and 14 days after first treatment, and changes of perfusion parameters were analyzed.

**Results:**

In treatment group, CEUS perfusion parameters showed a significant change. The peak enhancement (PE, 2.50 x10^3^±1.68 x10^3^ vs 5.55x10^2^±4.65x10^2^, p = 0.010) and wash-in and wash out AUC (_WiWo_AUC, 1.07x10^5^±6.48 x10^4^ vs 2.65x10^4^±2.25x10^4^, p = 0.009) had significantly decreased two weeks after treatment. On the contrary, control group did not show a significant change, including PE (1.15 x10^3^±7.53x10^2^ vs 9.43x10^2^± 7.81 x10^2^, p = 0.632) and _WiWo_AUC (5.09 x10^4^±3.25x10^4^ vs 5.92 x10^4^±3.20x10^4^, p = 0.646). For reproducibility, the various degrees of inter-scanner reproducibility were from poor to good (ICC: <0.01–0.63). However, inter-operator reproducibility of important perfusion parameters, including _Wi_AUC, _Wo_AUC, and _WiWo_AUC, ranged from fair to excellent (ICC: 0.59–0.93) in a different scanner.

**Conclusion:**

Our results suggest that CEUS is useful for assessment of the treatment response after targeted therapy and with fair to excellent inter-operator reproducibility.

## Introduction

Various imaging techniques are widely used for the diagnosis and monitoring of the treatment response of cancer. Among the available imaging modalities, contrast-enhanced ultrasound (CEUS) with microbubble contrast agents is an emerging imaging technique used for monitoring targeted treatments as it provides real-time qualitative and quantitative information regarding tumor vascularity with high sensitivity. CEUS also has the advantage of being a non-invasive method which can be performed bedside and allow repeated examinations without renal toxicity [1–3]. Although CEUS has strong points for evaluating the treatment response using quantitative information for tumor perfusion, there are only a few studies regarding CEUS reproducibility. Because of the need for standardization of CEUS findings and for defining inter-scanner and inter-operator reproducibility, there are some attempts to improve reproducibility and to standardize CEUS findings and protocols, such as the quantitative imaging biomarkers alliance (QIBA) which is a group of researchers, healthcare professionals, and industry personnel [4].

For hepatocellular carcinoma (HCC), one of the most well-known hypervascular tumors, angiogenesis has an important role regarding tumor growth and development. Therefore, angiogenesis is considered an important feature for tumor-targeted therapy [3, 5]. The recent treatment strategy of HCC was directed to the effect of targeted therapy, and sorafenib (Nexavar®, Bayer Health Care, Leverkusen, Germany) is one of the targeted therapies widely used. Sorafenib is a molecularly targeted, multi-kinase inhibitor that suppresses the signal transduction pathways that mediate tumor growth and angiogenesis [6]. It is known that reductions in intra-tumor blood flow before measurable change in tumor size with therapeutic response are often observed after molecularly targeted therapy [7–9]. Therefore, there is a need for better imaging features than tumor size in order to evaluate the efficacy of sorafenib therapy with successful reproducibility [10]. However, to date, there have been small number of studies focusing on the inter-scanner and inter-operator reproducibility using CEUS. The purpose of this study is to assess therapeutic responses monitoring after targeted therapy in an orthotopic rat model of HCC using CEUS while focusing on the inter-scanner and inter-operator reproducibility.

## Materials and methods

This study was approved by Seoul National University Hospital’s Institutional Animal Care and Use Committee (IACUC; No. 17-0125-S1A0) and was performed in accordance with the Guide for our IACUC and the National Institute of Health Guide for the Care and Use of Laboratory Animals.

### Animal models

Seventeen, male Sprague–Dawley rats (approximately two months old; body weight, 250–340 g) were used for the human HCC xenograft model. The N1-S1 rat hepatoma cells (CRL-1604; ATCC, Manassas, VA, USA) were cultured in RPMI-1640 (WelGENE, Daegu, Korea) supplemented with 10% fetal bovine serum (WelGENE) and a 1% penicillin/streptomycin mixture (Gibco, Grand Island, NY, USA) at 37°C in a humidified atmosphere containing oxygen and 5% CO_2_. After testing the viability with Trypan blue staining (confirming > 90% cell viability for each tumor implantation procedure), cells were harvested for implantation. Before every handling, all rats were anesthetised to minimize animal suffering and distress. Rats were housed in the standard animal care facility cage during all experiments, which kept on a natural dark and light (12:12 h) cycle, in a temperature and humidity-controlled room and had free access to food and rodent food pellets. To anesthetize the rats, a mixture of zolazepam (5 mg/kg, Zoletil®; Virbac, Carros, France) and xylazine (10 mg/kg, Rompun®; Bayer-Schering Pharma, Berlin, Germany) was intramuscularly injected into the hind limb of each rat. All rats then underwent an upper midline incision and their left hepatic lobes were exposed on a sterile compress. According to the established protocols for the N1-S1 tumor model [11, 12], a suspension of 5 x 10^6^ cells prepared in 50 μL of medium were slowly injected under the hepatic capsule into the left lobe of each rat using a 30-gauge needle. A gentle compression with handheld electrocautery (Bovie Medical Corporation, Clearwater, FL, USA) and cotton applicator were used for few seconds in order to avoid bleeding and reflux of the cells. To prevent spontaneous tumor regression of the N1-S1, cyclosporine A (20 mg/kg/day; Chong Kun Dang Pharmaceutical Corp., Seoul, Korea) was subcutaneously administrated from one day before the tumor implantation until four days after the surgery [13]. After the surgery, the animals were transferred to their home cage, which kept on a natural dark and light cycle in a temperature and humidity-controlled room. The rats were allowed access to food and pellets after recovery from anesthesia with strict observation for the first 2 hours after surgery. Rats were received daily postoperative care with regular food intake amount and body weight measured during the follow-up period after surgery. The rats had be planned euthanasia when a decrease in food intake for three days or weight loss of 20%

### Experimental protocol

**Fig 1:**
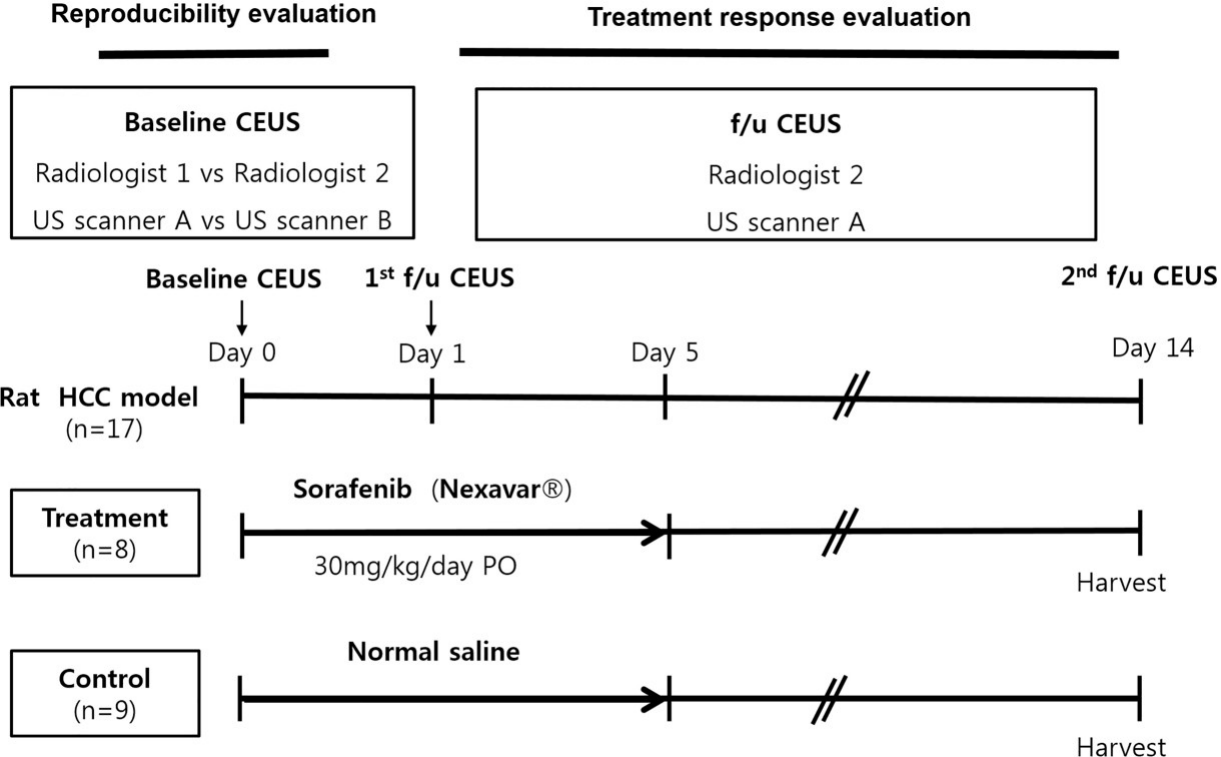
Outline of the experimental design. Note–CEUS: contrast enhanced ultrasound examination. f/u: follow up.

Two weeks following tumor implantation, an US examination was performed to check tumor growth. A CEUS examination and treatment were started only for rats with visible tumor having reached in diameter of approximately 10 mm. All of the examinations were performed after achieving anesthesia using the same method as that used for tumor implantation. For reproducibility evaluation, two radiologists (000 with 18 years of clinical experience in abdominal US, and 000 with five years of clinical experience in abdominal US) performed initial CEUS using two different US scanners, i.e. LOGIQ E9 ultrasound system (**US scanner A,** GE Healthcare, Wauwatosa, WI, USA), and RS80A ultrasound system (**US scanner B,** Samsung Medison, Seoul, Korea). Those two kind of scanners are widely used US scanner thesedays, and they were used in various previous CEUS imaging studies such as tumor detection and characterization, or tissue vascularization and perfusion [14–18]. After the CEUS examination, the rats were randomly divided into two groups as follows: (1) the sorafenib-treated group (n = 8); and (2) the control group (n = 9). In the sorafenib-treated group, sorafenib was administered orally at a dosage of 30 mg/kg/day for five days. The same volume of saline was administered orally to the control group rats. Follow up CEUS examinations were performed one day and 14 days after the first sorafenib administration by one radiologist (000) using US scanner A. After the last CEUS examination, all of the rats were sacrificed with a lethal dose of sodium pentobarbital (100 mg/kg body weight, intraperitoneal). Just after sacrifice, the tumors of each rat were collected for histologic analysis. The tumor length at the maximum dimension and the CEUS datasets of each rat were obtained in the same manner as with the initial CEUS examination. The study design is outlined in **Fig 1**.

### Contrast-enhanced ultrasound examination

**Fig 2:**
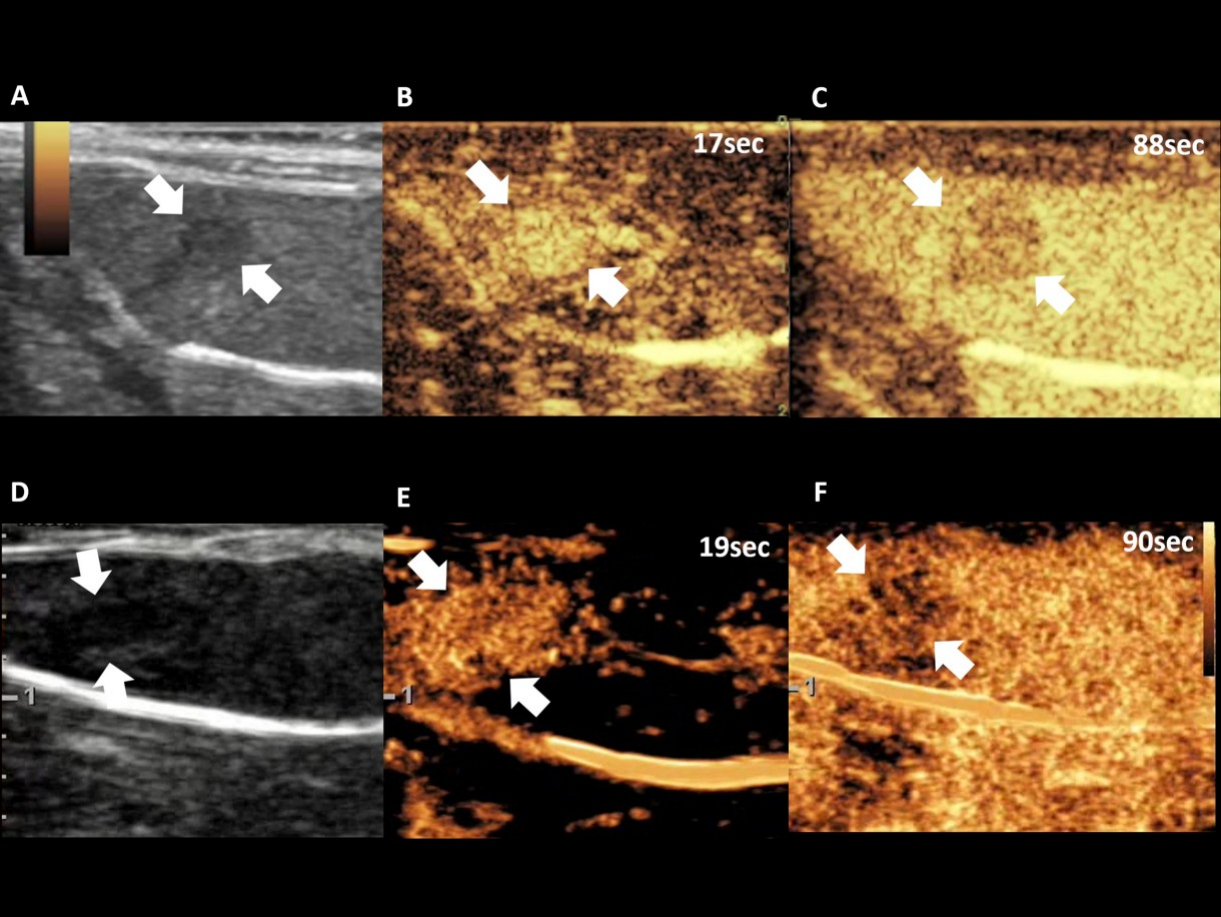
Representative cases of CEUS examination of US scanner A (A, B, C) and US scanner B (D, E, F), demonstrating B-mode imaging (A, D), arterial phase (B, E), and delayed phase (C, F). Note–CEUS: contrast enhanced ultrasound examination. Numbers in figure indicates time from contrast injection.

Two radiologists (000, and 000) conducted an ultrasound examination using a 2–9 MHz linear-array transducer of both US scanner A (9L-D, center frequency, 5.0 MHz, mechanical index (MI), 1.2 on B-mode imaging, and <0.2 on coded contrast imaging mode, dynamic range, 69 dB, gain, 70%, and US scanner B (LA2-9A, center frequency, 5.15 MHz, MI, 1.4 on B-mode imaging, and 0.07 on CEUS+ imaging mode, dynamic range, 35 dB, gain, 60%). Each operator obtained B-mode images and measured the tumor length at the maximum dimension before the CEUS examination. The SonoVue® (Bracco Suisse SA, Geneva, Switzerland) US contrast agent was used in this study. Cannulation was done in the tail vein of each rat with a 27-gauge needle. Bolus injection of 5 x 10^7^ microbubbles (0.1 mL) was done through the tail vein by hand injection following 0.1 mL of saline flush. After microbubble injection, the CEUS data were obtained continuously for 90 seconds in each rat by each operator **(Fig 2)**. For CEUS data acquisition, the coded contrast imaging with reduced MI (<0.2) was used by US scanner A, and CEUS+ with a MI level of 0.07 was used by US scanner B. The four, different contrast injections for each rat were performed at least 30 minutes apart in order to allow clearance of microbubbles from previous injections [19–21].

### Contrast-enhanced ultrasound imaging analysis

**Fig 3:**
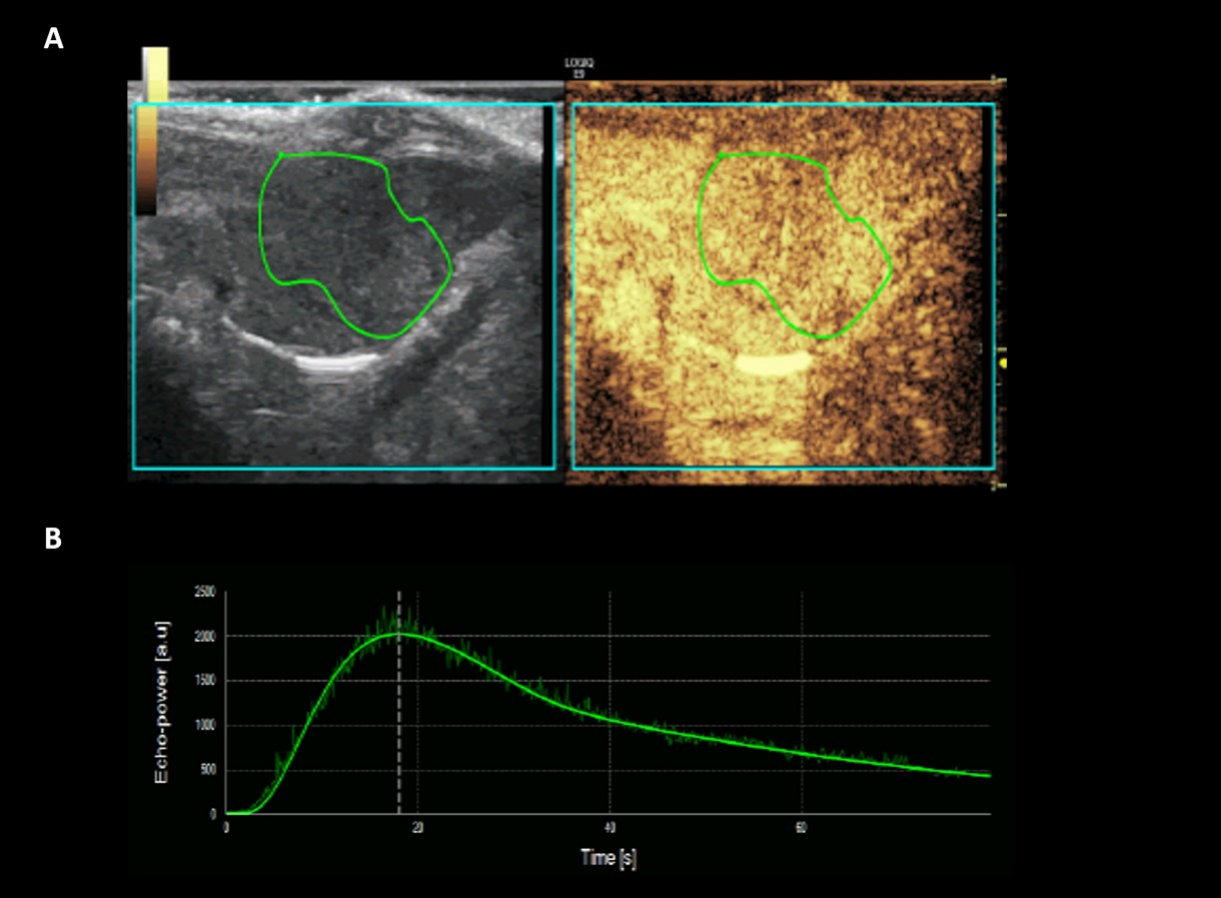
Representative case of CEUS image analysis using perfusion analysis software (VueBox ®), ROI manually drawn over tumor (A), and corresponding time-intensity curve profile (B). Note–CEUS: contrast enhanced ultrasound examination. ROI: region of interest.

One radiologist (000) analyzed all of the CEUS datasets of three sessions using perfusion analysis software (VueBox ®, Bracco Suisse SA, Geneva, Switzerland). Each cine clip of CEUS data was assessed, and the polygonal region of interest (ROI) covering the entire tumor was drawn manually on the image **(Fig 3)**. If there was a change in the tumor position due to respiratory motion during the scanning, the ROI was automatically calibrated by the software. When automatic calibration by software was insufficient, the ROI was adjusted manually. Time-intensity curves were automatically fitted to the log-normal model using the non-linear least-squares regression method. All of the following 11 perfusion parameters were automatically calculated: peak enhancement (PE); rise time; fall time; mean transit time (MTT); time to peak enhancement (TTP); wash-in rate (_Wi_R); washout rate (_Wo_R); wash-in area under the curve (_Wi_AUC); wash-out area under the curve (_Wo_AUC); wash-in and wash-out areas under the curve (_WiWo_AUC); and wash-in perfusion index (_Wi_PI). Changes of perfusion parameters were analyzed to evaluate the treatment effect [11–13].

### Histologic analysis

Tumor tissue was excised after the follow-up imaging study 14 days after the first sorafenib administration. Hematoxylin and eosin (H&E) stain was done to evaluate the morphologic features and the necrotic fraction of the tumor. The terminal, deoxynucleotidyl, transferase-mediated, dUTP nick end-labelling (TUNEL) staining using a detection kit (Millipore, Bedford, MA, USA) was performed to calculate the apoptosis index. The immunohistochemistry staining was performed using an antibody to CD31 (ab28364, 1:50 Abcam, Cambridge, UK) in order to obtain the mean vessel density (MVD). All stained tissue sections were scanned at x40 magnification by a Leica AS LMD laser microdissection microscope (Leica, Wetzlar, Germany) and saved as digital images (in Joint Photographic Experts Group format). Using Image J software (National Institutes of Health, Bethesda, MD, USA), the necrotic fraction (%), apoptosis index (%), and the MVD of each sample were calculated by one author (00) who was blinded to the treatment status of the rats. The necrotic fraction was calculated as the area of the largest cross-section of the entire tumor divided by the area of necrosis at lower-power field (x40). The apoptosis index was calculated as the averaged proportion of TUNEL-positive cells with respect to the total number of cells at high-power fields (x400) of five, random areas of each sample avoiding necrotic area. MVD was obtained by the maximum number of CD31-stained vessels among the randomly chosen five, higher vascular areas of each sample at high magnification (x200, 0.739 mm^2^) [22–25].

### Statistical analysis

The paired t-test was used to compare the perfusion parameters between two, different operators or two, different US scanners and to evaluate changes in the perfusion parameters between baseline and two weeks after in either the treatment or control group. The student t-test was used to compare perfusion parameters and histologic features between the treatment and control group. The Pearson correlation coefficient (r) was calculated between the perfusion parameters and the histologic features. For inter-scanner and inter-operator reproducibility, intra-class correlation coefficients (ICCs) with 95% confidence intervals (CIs) were obtained. An ICC of 0–0.39 indicated poor; 0.40–0.59, fair; 0.60–0.74, good; and 0.75–1.0, excellent agreement [26]. All statistical analyses were performed using commercially available software programs (SPSS version 21, SPSS, IBM, Armonk, NY, USA; or MedCalc version 18, MedCalc Software, Mariakerke, Belgium) and with a p-value less than 0.05 considered to indicate a statistically significant difference.

## Results

Among 17 of the tumor-implanted rats, one rat was excluded from the reproducibility evaluation because of poor tumor enhancement during one of four CEUS sessions due to compromised vessel integrity. In total, 16 rats were assessed for inter-scanner and inter-operator reproducibility. For the treatment response evaluation, a total of 17 rats, including the treatment group (n = 8), and the control group (n = 9), were assessed. The average size of all tumors included in this study was 11.1±3.69 mm and the maximum size of tumors was 18.0 mm.

### Inter-scanner reproducibility evaluation of each perfusion parameter

**Table 1** summarizes the inter-scanner reproducibility of the perfusion parameters with two operators. There was a significant variation of the perfusion parameters between the two, different US scanners. Except for the mean transit time, all of the perfusion parameters obtained by the two, different US scanners demonstrated significant differences for at least one operator. The pairwise ICC of 11 perfusion parameters for two, different scanners ranged from <0.01 to 0.63. The pairwise ICC of the major perfusion parameters, such as _Wi_AUC (0.01 and <0.01), _Wo_AUC (0.01, and 0.02), and _WiWo_AUC (0.01 and 0.02), demonstrated poor agreement.

**Table 1:** Comparison with inter-scanner reproducibility of perfusion parameters obtained by two operators.

		US scanner A	US scanner B	P	ICC
**PE (a.u)**	**R1**	1.30x10^3^ ± 9.31x10^2^	1.63x10^5^ ± 2.67x10^5^	0.028	<0.01(-0.99,0.59)
	**R2**	1.64x10^3^ ± 1.43x10^3^	5.25x10^4^ ± 4.32x10^4^	0.028	0.03(-0.35,0.46)
_**Wi**_**AUC (a.u)**	**R1**	1.43x10^4^ ± 1.05x10^4^	6.26x10^5^ ± 7.71x10^5^	0.006	0.01(-0.69,0.55)
	**R2**	1.81x10^4^ ± 1.34x10^4^	5.73x10^5^ ± 8.05x10^5^	0.894	<0.01 (-0.86,0.57)
**RT (sec)**	**R1**	16.8 ± 7.81	9.68 ± 5.50	0.010	<0.01 (-0.84,0.54)
	**R2**	17.3 ± 7.17	9.49 ± 7.51	0.002	0.63(-0.25,0.89)
**MTT (sec)**	**R1**	1.64x10^2^ ± 1.69	86.4 ± 83.8	0.083	0.31(-0.64,0.74)
	**R2**	1.58x10^2^ ± 1.61	1.47x10^2^ ± 1.85	0.208	0.51(-0.48,0.83)
**TTP (sec)**	**R1**	20.4 ± 9.26	15.8 ± 9.72	0.200	<0.01 (-2.00,0.59)
	**R2**	21.2 ± 8.40	12.4 ± 9.45	0.004	0.62(-0.22,0.88)
_**Wi**_**R (a.u)**	**R1**	1.47x10^2^ ± 93.9	4.18x10^5^ ± 9.69x10^5^	0.106	<0.01 (-1.38,0.62)
	**R2**	1.86x10^2^ ± 2.06	9.85x10^3^ ± 1.08x10^4^	<0.001	0.03(-0.55,0.53)
_**Wi**_**PI (a.u)**	**R1**	8.71x10^2^ ± 6.17x10^2^	1.00x10^5^ ± 1.67x10^5^	0.031	<0.01 (-1.01,0.59)
	**R2**	1.10x10^3^ ± 9.59x10^2^	3.44x10^4^ ± 2.79x10^4^	0.037	0.03(-0.34,0.46)
_**Wo**_**AUC (a.u)**	**R1**	4.20x10^4^ ± 3.15x10^4^	1.50x10^6^ ± 1.71x10^6^	0.004	0.01(-0.63,0.53)
	**R2**	6.25x10^4^ ± 6.08x10^4^	1.08x10^6^ ± 1.81x10^6^	0.558	0.02(-1.04,0.60)
_**WiWo**_**AUC (a.u)**	**R1**	5.63x10^4^ ± 4.18x10^4^	2.21x10^6^ ± 2.48x10^6^	0,003	0.01(-0.61,0.53)
	**R2**	8.05x10^4^ ± 7.19x10^4^	1.52x10^6^ ± 2.45x10^6^	0.492	0.02(-0.99,0.60)
**FT (sec)**	**R1**	52.6±25.0	23.9±17.4	0.001	0.24(-0.31,0.65)
	**R2**	53.2±25.6	27.3±24.7	0.010	0.58(-0.23,0.86)
_**Wo**_**R (a.u)**	**R1**	31.5±25.6	1.60x10^4^ ± 3.57x10^4^	0.094	<0.01(-1.34,0.62)
	**R2**	40.6±42.7	3.55x10^3^ ± 5.00x10^3^	<0.001	0.02(-0.80,0.57)

Note. − A: GE LOGIQ E9. B: Samsung RS80A. PE: peak enhancement. _Wi_AUC: Wash-in area under the curve. RT: Rise time. MTT: mean transit time. TTP: Time to peak. _Wi_R: Wash-in rate. _Wi_PI: Wash-in Perfusion Index. _Wo_AUC: Wash-out area under the curve. _WiWo_AUC: Wash-in and Wash-out area under the curve. FT: Fall Time. _Wo_R: Wash-out rate. a.u: arbitrary units.R1: Radiologist 1, R2: Radiologist 2.

### Inter-operator reproducibility evaluation of each perfusion parameters

There was no significant difference in each of the perfusion parameters between the two operators when using the same US scanner. Although the pairwise ICC of 11 perfusion parameters for two, different operators ranged widely from <0.01 to 0.93, the pairwise ICC of the major perfusion parameters, such as _Wi_AUC (0.76 and 0.93), _Wo_AUC (0.59 and 0.90), and _WiWo_AUC (0.65 and 0.88), showed fair to excellent agreement. The inter-operator reproducibility of all of the perfusion parameters is noted in **Table 2.**

**Table 2:** Comparison with inter-operator reproducibility of perfusion parameters using two kinds of US scanners.

	US scanner	Radiologist 1	Radiologist 2	P	ICC
**PE (a.u)**	**A**	1.30x10^3^ ±9.31x10^2^	1.64x10^3^ ± 1.43x10^3^	0.277	**0.65(0.04,0.88)**
	**B**	1.63x10^5^ ± 2.67x10^5^	5.25x10^4^ ± 4.32 x10^4^	0.088	**0.30(-0.68,0.74)**
_**Wi**_**AUC (a.u)**	**A**	1.43x10^4^ ± 1.05x10^4^	1.81x10^4^ ± 1.34x10^4^	0.163	**0.76(0.36,0.92)**
	**B**	6.26x10^5^ ± 7.71x10^5^	5.73x10^5^ ± 8.05x10^5^	0.620	**0.93(0.79,0.97)**
**RT (sec)**	**A**	16.8±7.81	17.3 ± 7.17	0.772	0.77(0.33,0.92)
	**B**	9.68±5.50	9.49 ± 7.51	0.936	<0.01(-2.40,0.65)
**MTT (sec)**	**A**	1.64x10^2^ ± 1.69x10^2^	1.58x10^2^ ± 1.61x10^2^	0.876	0.66(-0.02,0.88)
	**B**	86.4 ± 83.8	1.47x10^2^ ± 1.85x10^2^	0.276	<0.01(-2.47,0.56)
**TTP (sec)**	**A**	20.4 ± 9.26	21.2±8.40	0.698	0.73(0.21,0.91)
	**B**	15.8 ± 9.72	12.4±9.45	0.370	<0.01(-3.04,0.53)
_**Wi**_**R (a.u)**	**A**	1.47x10^2^ ± 93.9	1.86x10^2^ ± 2.06x10^2^	0.410	0.52(-0.37,0.83)
	**B**	4.18x10^4^ ± 9.69x10^4^	9.85x10^3^ ± 1.08x10^4^	0.205	0.05(-1.51,0.66)
_**Wi**_**PI (a.u)**	**A**	8.71x10^2^ ± 6.17x10^2^	1.10x10^3^ ± 9.59x10^2^	0.275	0.66(0.06,0.88)
	**B**	1.00x10^5^ ± 1.67x10^5^	3.44x10^4^ ± 2.79x10^4^	0.098	0.33(-0.63,0.75)
_**Wo**_**AUC (a.u)**	**A**	4.20x10^4^ ± 3.15x10^4^	6.25x10^4^ ± 6.08x10^4^	0.130	**0.59(-0.07,0.85)**
	**B**	1.50x10^6^ ± 1.71x10^6^	1.08x10^6^ ± 1.81x10^6^	0.125	**0.90(0.72,0.97)**
_**WiWo**_**AUC (a.u)**	**A**	5.63x10^4^ ± 4.18x10^4^	8.05x10^4^ ± 7.19x10^4^	0.118	**0.65(0.07,0.87)**
	**B**	2.21x10^6^ ± 2.48x10^6^	1.52x10^6^ ± 2.45x10^6^	0.099	**0.88(0.65,0.96)**
**FT (sec)**	**A**	52.6 ± 25.0	53.1 ± 25.6	0.922	0.80(0.40,0.93)
	**B**	23.4 ± 17.4	27.3 ± 24.7	0.668	<0.01(-2.73,0.61)
_**Wo**_**R (a.u)**	**A**	31.4 ± 25.6	40.6 ± 42.7	0.414	0.39(-0.77,0.79)
	**B**	1.60x10^4^ ± 3.57x10^4^	3.55x10^3^ ± 5.00x10^3^	0.183	0.04(-1.47,0.66)

Note. − A: GE LOGIQ E9. B: Samsung RS80A. PE: peak enhancement. _Wi_AUC: Wash-in area under the curve. RT: Rise time. MTT: mean transit time. TTP: Time to peak. _Wi_R: Wash-in rate. _Wi_PI: Wash-in Perfusion Index. _Wo_AUC: Wash-out area under the curve. _WiWo_AUC: Wash-in and Wash-out area under the curve. FT: Fall Time. _Wo_R: Wash-out rate. a.u: arbitrary units.

### Therapeutic responses evaluation using CEUS after Sorafenib treatment

**Fig 4:**
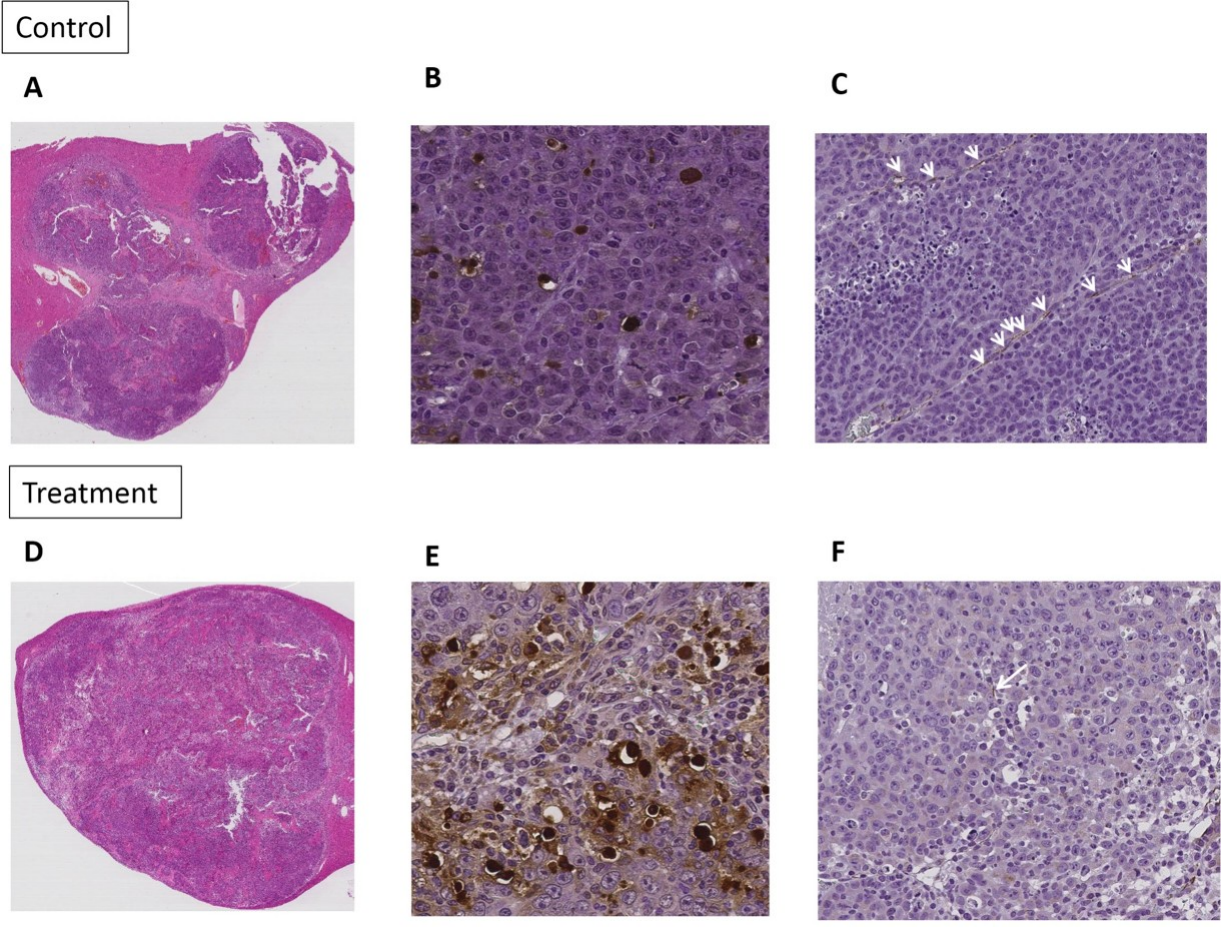
Representative cases of pathologic analysis, H&E (x40) (A and D), TUNEL (x400) (B and E), CD31 assay (x200) (C and F) of control group and sorafenib treated group. Note–H&E: hematoxylin and eosin stain. TUNEL: terminal deoxynucleotidyl transferase-mediated dUTP nick end-labelling staining.

In the histologic analysis, there were significant differences in the necrotic fraction, apoptosis index, and MVD of the tumor between the treatment and the control groups. The treatment group showed significantly larger necrotic area (47.7% ± 6.87 vs. 31.3% ± 10.8), higher apoptotic cell counts (49.1% ± 15.0 vs. 16.1% ± 7.15), and fewer microvessels (0.53 ± 0.35 vs. 1.76 ± 0.71) within the tumor than the control group (p <0.05, **Fig 4)**. **Table 3** summarizes the comparison of the histologic features between the treatment and the control groups. In the CEUS examination, the tumor size had significantly increased after two weeks in both treatment groups (9.75mm ± 3.77 vs 13.9mm ± 7.72, *P* = 0.032) and the control group (12.3mm ± 3.35 vs 26.7mm ±9.15, *P* = 0.003). However, the changes of perfusion parameters between the two groups were different. In the treatment group, PE (2.50 x 10^3^a.u ± 1.68x10^3^ vs 5.55 x 10^2^a.u ± 4.65 x 10^2^), _Wi_AUC (2.70 x 10^4^ a.u ± 1.65 x 10^4^ vs 6.61 x 10^3^ a.u ± 5.57 x 10^3^), _Wo_AUC (8.00 x 10^4^ a.u ± 4.83 x 10^4^ vs 1.99x10^4^ a.u ±1.71 x 10^4^), and _WiWo_AUC (1.07 x 10^5^ a.u ± 6.48 x 10^4^ vs 2.65 x 10^4^ a.u ± 2.25 x 10^4^) were significantly decreased (p <0.05). On the contrary, in the control group, PE, _Wi_AUC, _Wo_AUC, and _WiWo_AUC showed no significant change after two weeks.

**Table 3:** Comparison with histologic features between sorafenib treatment and control group.

	Treatment (n = 8)	Control (n = 9)	*P*
**Necrotic fraction (%)**	47.7±6.87	31.3±10.8	0.002
**Apoptosis index (%)**	49.1±15.0	16.1±7.15	<0.001
**Microvessel density (/0.739mm**^**2**^**)**	0.53±0.35	1.76±0.71	0.001

There was no correlation between the histologic features and the perfusion parameters, including the tumor size, PE, _Wi_AUC, _Wo_AUC, and _WiWo_ AUC (**S1 Table).** In addition, _Wi_R (2.92 x 10^2^a.u ± 2.09 x 10^2^ vs 53.4a.u ± 44.2), _Wi_PI (1.72 x 10^3^a.u ± 1.08 x 10^3^ vs 3.71 x 10^2^a.u ± 3.10 x 10^2^), and _Wo_R (55.0a.u ± 36.9 vs 12.1a.u ±10.4) were significantly decreased in the treatment group (p <0.05), and the rise time (17.8sec ± 7.09 vs 26.1sec ± 8.88, *P* = 0.040) was significantly increased in the control group **(Table 4)**.

**Table 4:** Comparison with tumor size and perfusion parameter of baseline, and two weeks after treatment.

	Control			Treatment		
	Baseline	Two weeks	P	Baseline	Two weeks	P
**Size (mm)**	**12.3±3.35**	**26.7±9.15**	**0.003**	**9.75±3.77**	**13.9±7.72**	**0.032**
**PE (a.u)**	1.15 x10^3^±7.53x10^2^	9.43 x10^2^± 7.81 x10^2^	0.632	**2.50 x10**^**3**^**±1.68 x10**^**3**^	**5.55x10**^**2**^**±4.65 x10**^**2**^	**0.010**
_**Wi**_**AUC (a.u)**	1.26 x10^4^±7.86x10^3^	1.32 x10^4^±7.39 x10^3^	0.875	**2.70 x10**^**4**^**±1.65 x10**^**4**^	**6.61 x10**^**3**^**±5.57 x10**^**3**^	**0.009**
**RT (sec)**	**17.8±7.09**	**26.1±8.88**	**0.040**	16.0±3.87	17.1±4.38	0.460
**MTT (sec)**	2.64 x10^2^±2.26 x10^2^	2.79 x10^2^±2.12 x10^2^	0.870	1.11 x10^2^±7.21 x10^2^	13.4±74.1	0.432
**TTP (sec)**	22.0±9.08	34.4±18.8	0.059	18.6±5.08	20.4±5.25	0.312
_**Wi**_**R (a.u)**	2.19 x10^2^±3.68 x10^2^	95.6±130	0.401	**2.92 x10**^**2**^**±2.09x10**^**2**^	**53.4±44.2**	**0.015**
_**Wi**_**PI (a.u)**	7.90 x10^2^±5.43x10^2^	6.50 x10^2^±5.66x10^2^	0.651	**1.72 x10**^**3**^**±1.08 x10**^**3**^	**3.71x 10**^**2**^**±3.10x10**^**2**^	**0.010**
_**Wo**_**AUC (a.u)**	3.83 x10^4^±2.54 x10^4^	4.60 x10^4^±2.55x10^4^	0.592	**8.00 x10**^**4**^**±4.83 x10**^**4**^	**1.99 x10**^**4**^**±1.71 x10**^**4**^	**0.009**
_**WiWo**_**AUC (a.u)**	5.09 x10^4^±3.25x10^4^	5.92 x10^4^±3.20x10^4^	0.646	**1.07x10**^**5**^**±6.48 x10**^**4**^	**2.65 x10**^**4**^**±2.25x10**^**4**^	**0.009**
**FT (sec)**	54.5±19.6	88.1±43.3	0.055	52.3±10.0	51.9±14.7	0.867
_**Wo**_**R (a.u)**	25.4±17.9	14.6±13.3	0.184	**55.0±36.9**	**12.1±10.4**	**0.015**

Note—PE: peak enhancement. _Wi_AUC: Wash-in area under the curve. RT: Rise time. MTT: mean transit time. TTP: Time to peak. _Wi_R: Wash-in rate. _Wi_PI: Wash-in Perfusion Index. _Wo_AUC: Wash-out area under the curve. _WiWo_AUC: Wash-in and Wash-out area under the curve. FT: Fall Time. _Wo_R: Wash-out rate. a.u: arbitrary units.

## Discussion

In our study, CEUS was useful in the therapeutic response evaluation after targeted therapy using perfusion parameters such as PE, _Wi_AUC, _Wo_AUC, and _WiWo_AUC. Inter-operator reproducibility was fair and excellent in _WiWo_AUC (ICC: 0.65 and 0.88) _Wi_AUC (ICC: 0.76 and 0.93), and _Wo_AUC (ICC: 0.59 and 0.90). However, various degrees of inter-scanner reproducibility, from poor to good (ICC: <0.01–0.63), were observed. Our results support the low inter-scanner exchangeability although acceptable inter-operator exchangeability of CEUS in perfusion parameters.

Standardization of CEUS perfusion parameters is essential for the clinical application. Reliable and reproducible quantification of the imaging biomarkers during follow-up is necessary for making a proper treatment plan for patients. In response to this need for standardization of the imaging biomarkers, the Radiological Society of North America organized the QIBA with the mission of improving the value and practicality of quantitative imaging biomarkers by reducing the variability across devices, patients, and time [27]. However there are only a few studies regarding the reproducibility of CEUS perfusion parameters in terms of inter-scanner and inter-operator. Zocco et al. investigated intra-observer reproducibility for the entire CEUS process after sorafenib treatment in HCC patients. The mean coefficient of variation (CV) of PE, AUC, TTP, and MTT ranged from 4.7% to 9.7% and is considered low-variance. The kappa values of five perfusion parameters were between 0.82 and 0.95, thus suggesting good to excellent agreement [10]. Ghulam et al. demonstrated excellent intra and inter-operator variability in measuring intraluminal thrombus volume (ICC: 0.95–0.99 and 0.92–0.98) and thickness (ICC: 0.86–0.94 and 0.88–0.98) of abdominal aortic aneurysms with 3D-CEUS [28]. Zoppellaro et al also reported good in intra‐operator (ICC: 0.929, p<0.001) and inter‐operator reproducibility (ICC: 0.926) of CEUS in myocardial contrast echocardiography [29]. However, they did not evaluate the inter-scanner reproducibility during the CEUS examination. In our study, the pairwise ICC of the inter-operator reproducibility during the CEUS examination for major perfusion parameters, including PE, _Wi_AUC, _Wo_AUC, and _WiWo_AUC, ranged from 0.59 to 0.90 for at least one scanner and thus suggesting fair to excellent agreement. However, the inter-scanner reproducibility of the four, major perfusion parameters were between <0.01 and 0.02, which were considered to be poor reproducibility. We believe this inter-scanner variability could be attributed to a number of systemic factors. According to recently published paper by Averkiou et al, one of the cause of the inter-scanner variability in especially of amplitude parameters such as PE and AUC is arbitrarily assigned absulute amplitude values by scanner developers. They also demonstrated much lower inter-scanner variability of time parameters such as RT and MTT than amplitude parameteres [30]. Different dynmaic range among scanners can also cause inter-scanner variability, because linearized log-compressed data are finally converted to arbitrary unit data by application of dyanmic range. In our study, we used default dynamic range optimized by the vendors, which were different from two scannars (69 and 35 dB). In addition, different mechanical index can result inter-scanner variability. Tang et al showed an increase of approximately 50 percent in the normalized echo signal power would be expected if the MI changes from 0.05 to 0.1 [4]. Finally, different central frequency of probe can cause inter-scanner variability due to high dependency of microbubble contrast agents on ultrasound frequency [31]. Our experience suggests that a CEUS examination with different US scanners during follow-up may provide incorrect information regarding the treatment response.

As CEUS provides quantitative tumor perfusion parameters with high sensitivity, this allows prediction of the early treatment response after targeted therapy [32]. After bolus injection of contrast microbubbles, a time-intensity curve showing average intensity in an ROI as a function of time is formed. Several perfusion parameters are derived from the time-intensity curve, and some parameters, such as PE and AUC, are more correlated to the local blood volume of the region, while other parameters, such as TTP and the rise time, are more correlated to blood flow. Therefore, the decrease in PE and AUC correlates to the reductions in tumor blood volume [1, 23, 32, 33]. Based on this fact, there are several previous studies. Lassau et al. demonstrated the significant correlation between perfusion parameters and treatment response after bevacizumab treatment in HCC patients. They demonstrated decreases in _WiWo_AUC, _Wi_AUC, _Wo_AUC, and TTP measured three days after treatment correlated with the *Response evaluation criteria in solid tumors* (*RECIST*) response at two months following treatment (*P* < 0.05) [24]. Zocco et al. also demonstrated the significant correlation between CEUS perfusion parameters and the treatment response in 28, advanced HCC patients treated with sorafenib. They demonstrated the percentage changes in PE, _WiWo_AUC, and _Wi_R measured 15 days after treatment correlated with the RECIST response at two months (*P* < 0.05) [10]. In our study, perfusion parameters including PE (*P* = 0.01), _Wi_AUC (*P* = 0.009), _Wi_R, _Wi_PI, _Wo_AUC, _WiWo_AUC, and _Wo_R demonstrated a significant decrease in the treatment group two weeks after sorafenib treatment (*P* < 0.05), although there was no significant change in the control group. In addition, more necrotic areas, more apoptotic cells, and fewer microvessels in the treatment group were confirmed in histologic analysis (*P*<0.001–0.002). These results agree with those of previous studies.

There are some limitations to our study. First, we manually injected microbubble contrast agent into each rat. Although all injections were performed by one person, this could affect the variability of the CEUS parameters. Second, the N1-S1 rat hepatoma tumors have a property of spontaneous regression and we used an immunosuppressant to prevent spontaneous regression of the tumor [22]. As the immunosuppressant may affect tumor vascularity, further studies regarding the effects of the immunosuppressant on tumor vascularity will be required. Third, this study was conducted with rats and the number of rats included in the study was small. Therefore, there is a limitation for applying the results directly to the human population. Furthermore, evaluation with a large population would clarify the results. Finally, we did not evaluate intra- or inter-observer variability during the CEUS imaging analysis using perfusion analysis software.

In conclusion, our results suggest that CEUS is useful for assessment of the treatment response after targeted therapy with fair to excellent inter-operator reproducibility. However, significant inter-scanner variability was observed. Therefore, the perfusion parameters obtained by different US scanners should not be used interchangeably in longitudinal follow-up.

## Supporting information

S1 Table Correlation coefficient for evaluation of an association between imaging parameters and histologic features. (DOCX)
