# Supplementary material for: Therapeutic response monitoring after targeted therapy in an orthotopic rat model of hepatocellular carcinoma using contrast-enhanced ultrasound: Focusing on inter-scanner, and inter-operator reproducibility
Source: PLoS One. 2020 Dec 23;15(12):e0244304. doi: 10.1371/journal.pone.0244304 (PMC7757904; doi:10.1371/journal.pone.0244304)
Supplement: S1 Table — (DOCX) [file pone.0244304.s001.docx]

**S1 Table. Correlation coefficient for evaluation of an association between imaging parameters and histologic features.**

|  | **change in each value between baseline and two weeks after treatment** | | | | | | | |
| --- | --- | --- | --- | --- | --- | --- | --- | --- |
|  | **Tumor size** | **PE** | **WiAUC** | **WiR** | **WiPI** | **WoAUC** | **WiWoAUC** | **WoR** |
| **Necrotic fraction** | -0.6387 (P=0.0883) | -0.2787 (P=0.5039) | -0.083642 (P=0.8439) | -0.4417 (P=0.2732) | -0.2832 (P=0.4967) | -0.1307 (P=0.7578) | -0.1188 (P=0.7793) | -0.3997 (P=0.3265) |
| **Apoptosis index** | -0.4429 (P=0.2718) | -0.3660 (P=0.3726) | -0.2275 (P=0.5879) | -0.4749 (P=0.2343) | -0.3675 (P=0.3705) | -0.2245 (P=0.5930) | -0.2257 (P=0.5910) | -0.4613 (P=0.2500) |
| **MVD** | 0.4411 (P=0.2740) | 0.2046 (P=0.6270) | 0.1272 (P=0.7640) | 0.2549 (P=0.5423) | 0.2064 (P=0.6238) | 0.1563 (P=0.7118) | 0.1491 (P=0.7246) | 0.2437 (P=0.5608) |

Note - PE: peak enhancement. WiAUC: Wash-in area under the curve. RT: Rise time. MTT: mean transit time. . TTP: Time to peak. WiR: Wash-in rate. WiPI: Wash-in Perfusion Index. WoAUC: Wash-out area under the curve. WiWoAUC: Wash-in and Wash-out area under the curve. FT: Fall Time. WoR: Wash-out rate.
